# Sourcing data on chemical properties and hazard data from the US-EPA CompTox Chemicals Dashboard: A practical guide for human risk assessment

**DOI:** 10.1016/j.envint.2021.106566

**Published:** 2021-04-29

**Authors:** Antony J. Williams, Jason C. Lambert, Kris Thayer, Jean-Lou C.M. Dorne

**Affiliations:** a Center for Computational Toxicology and Exposure, Office of Research and Development, U.S. Environmental Protection Agency (U.S. EPA), Research Triangle Park, NC, USA; b Center for Public Health and Environmental Assessment, Office of Research and Development, U.S. Environmental Protection Agency (U.S. EPA), Research Triangle Park, NC, USA; c Scientific Committee and Emerging Risks Unit, Department of Risk Assessment and Scientific Assistance, European Food Safety Authority, 43126 Parma, Italy

**Keywords:** Computational toxicology, Human health, Risk assessment

## Abstract

For the past six decades, human health risk assessment of chemicals has relied on *in vivo* data from human epidemiological and experimental animal toxicological studies to inform the derivation of cancer and non-toxicity values. The ongoing evolution of this risk assessment paradigm in an environmental landscape of data-poor chemicals has highlighted the need to develop and implement non-testing methods, so-called New Approach Methodologies (NAMs). NAMs include a growing number of *in silico* and *in vitro* data streams designed to inform hazard properties of chemicals, including kinetics and dynamics at different levels of biological organization, environmental fate and transport, and exposure. NAMs provide a fit-for-purpose science-basis for human hazard and risk characterization of chemicals ranging from data-gap filling applications to broad evidence-based decision-making. Systematic assembly and delivery of empirical and predicted data for chemicals are paramount to advancing chemical evaluation, and software tools serve an essential role in delivering these data to the scientific community. The CompTox Chemicals Dashboard (from here on referred to as the “Dashboard”) is one such tool and is a publicly available web-based application developed by the US Environmental Protection Agency to provide access to chemistry, toxicity and exposure information for ~900,000 chemicals. The Dashboard is increasingly becoming a valuable resource for assessors tasked with the evaluation of potential human health risks associated with chemical exposures. In this context, the significant amount of information present in the Dashboard facilitates: 1) assembly of information on physicochemical properties and environmental fate and transport and exposure parameters and metrics; 2) identification of cancer and non-cancer health effects from extant human and experimental animal studies in the public domain and/or information not available in the public domain (i.e., “grey literature”); 3) systematic literature searching and review for developing cancer and non-cancer hazard evidence bases; and 4) access to mechanistic information that can aid or augment the analysis of traditional toxicology evidence bases, or potentially, serve as the primary basis for informing hazard identification and dose–response when traditional bioassay data are lacking. Finally, *in silico* predictive tools developed to conduct structure–activity or read-across analyses are also available within the Dashboard. This practical tutorial is intended to address key questions from the human health risk assessment community dealing with chemicals in both food and in the environment. Perspectives for future development or refinement of the Dashboard highlight foreseen activities to further support the research and risk assessment community in cancer and non-cancer chemical evaluations.

## 1. Introduction

Chemical risk assessment is an applied discipline that aims to determine levels of human or ecological exposures to substances in environmental media such as food, air, and waterthat should not result in substantial risk of toxicity (e.g., Acceptable Daily Intake, Reference Dose, Minimal Risk Level, etc.), and compare them with actual exposure levels (exposure assessment) to determine the risk for human or ecological (sub-)populations (risk characterization). Since the 1960s, hazard identification and dose–response assessment in the traditional human health risk assessment paradigm have primarily relied upon guideline-type (e.g., Organisation for Economic Co-operation and Development (OECD)) short-term, sub-chronic or chronic toxicology studies in relevant test species (e.g., rat, mouse, rabbit or dog). Traditional evaluation of the landscape of non-cancer or cancer effects across dose and time involves identification of apical/phenotypic “critical” effects to serve as the basis for candidate points of departure (PODs), including Lowest or No-Observed–Adverse-Effect-Level (LOAEL/NOAEL) and Benchmark Dose Levels (e.g., BMDL 1SD, BMDL_10_). For non-cancer effects, the PODs are typically divided by uncertainty or safety factors to account for extrapolations or data gaps in knowledge such as animal-to-human, human interindividual variability, use of a LOAEL (when a BMDL or NOAEL are not available), use of subchronic duration studies for chronic derivation, and/or limitations in the available hazard and dose–response database. This traditional assessment approach is applied broadly and underpins the derivation of values such as reference doses, acceptable daily intakes for regulated products (pesticides, food additives, food contact materials), Upper Limits (UL) for vitamins and minerals, tolerable daily intakes (TDI) for food and drug contaminants, or derived no-effect levels (DNELs) for industrial chemicals under the REACH (Registration, Evaluation, Authorization and Restriction of Chemicals) regulation (ECHA REACH, 2020). In the context of traditional cancer risk assessment, health outcomes such as tumor incidence or other neoplastic growth metrics are used to identify PODs. Such PODs have been used to derive oral or inhalation cancer slope factors (U.S.) or margins-of-exposure (Europe) along with weight-of-evidence narratives offering mechanistic interpretations (e.g., DNA reactivity; epigenetics; decreased immune surveillance; etc.) of observed tumorigenicity or neoplasia. The non-cancer and cancer risk assessment processes abbreviated here are highly resource and time intensive, dependent on diverse and often disparate data sources, and are typically applied to data-rich chemicals. Further, most legacy and emerging chemicals found in commerce and environmental media are relatively data-poor. As such, development of comprehensive, centralized, and flexible data platforms and tools for diverse fit-for-purpose applications are of paramount importance for advancing the science of risk assessment.

Over the past decade, the US EPA has developed the CompTox Chemicals Dashboard (Williams, 2017; Dashboard, 2020) as a resource to centralize access to a broad landscape of risk assessment relevant information for ~ 900,000 chemicals. The physicochemical, fate/transport/exposure, hazard, and dose–response information and analysis tools available in the Dashboard offer an optimal starting point for any problem formulation associated with the evaluation of chemical risks to human or ecological health, from initial screening and prioritization of groups of chemicals, to comprehensive risk assessment of single chemicals. The flexibility and modularity of the Dashboard facilitates diverse gradations of data assembly, export, visualization, and use ranging from targeted data gap filling to surfacing of all empirical and predicted data that may inform the entirety of the human health risk assessment paradigm (i.e., hazard identification, dose–response assessment, exposure assessment, and hazard characterization) for a given chemical. Specifically, the Dashboard can be utilized to locate information pertinent to conducting comprehensive literature searches, assemble information on chemical properties, and identify health information not available in the public domain (i.e., “grey literature”). The Dashboard is also the primary EPA platform for distribution of the high-throughput screening (HTS) results associated with the influential ToxCast and Tox21 research programs (Toxicity Forecasting: Advancing the Next Generation of Chemical Evaluation, 2020; Testing, 2020). This information may aid in the analysis or augment the interpretation of animal bioassay or epidemiological evidence, or potentially serve as the primary basis for reaching assessment conclusions when traditional repeat-dose study data are lacking. In addition to HTS data, the Dashboard provides opportunities to evaluate data-poor chemicals using structure–activity/read-across, thus leveraging information across multiple similarity contexts (e.g., structure, physicochemical properties, ADME [absorption, distribution, metabolism, and excretion], bioactivity/toxicity) between data-poor chemicals and more well characterized chemicals. This tutorial is intended to illustrate how the Dashboard can serve as a fundamental resource for data integration that is critical for human health or ecological assessment of chemicals.

## 2. Searching the Dashboard for chemical properties

The Dashboard can be searched by multiple chemical identifiers (e.g., chemical name(s), and Chemical Abstract Services Registry number or CAS RN), consumer product category (i.e., view chemicals found in certain product types), and assays/genes associated with HTS data. Data disseminated via the Dashboard are compiled from multiple sources, including: the EPA’s computational toxicology research databases and other agency databases such as the ECOTox Knowledgebase (EPA ECOTox Knowledgebase, 2020); multiple public domain databases (e.g., PubChem (PubChem, 2020), European Chemical Agency chemical dossiers (Agency, 2020); and the peer-reviewed literature. This tutorial is not intended to be exhaustive and the reader is pointed to the first Dashboard article (Williams, 2017) and the online Help file for further details (Dashboard and online help, 2020).

**Fig. 1.**
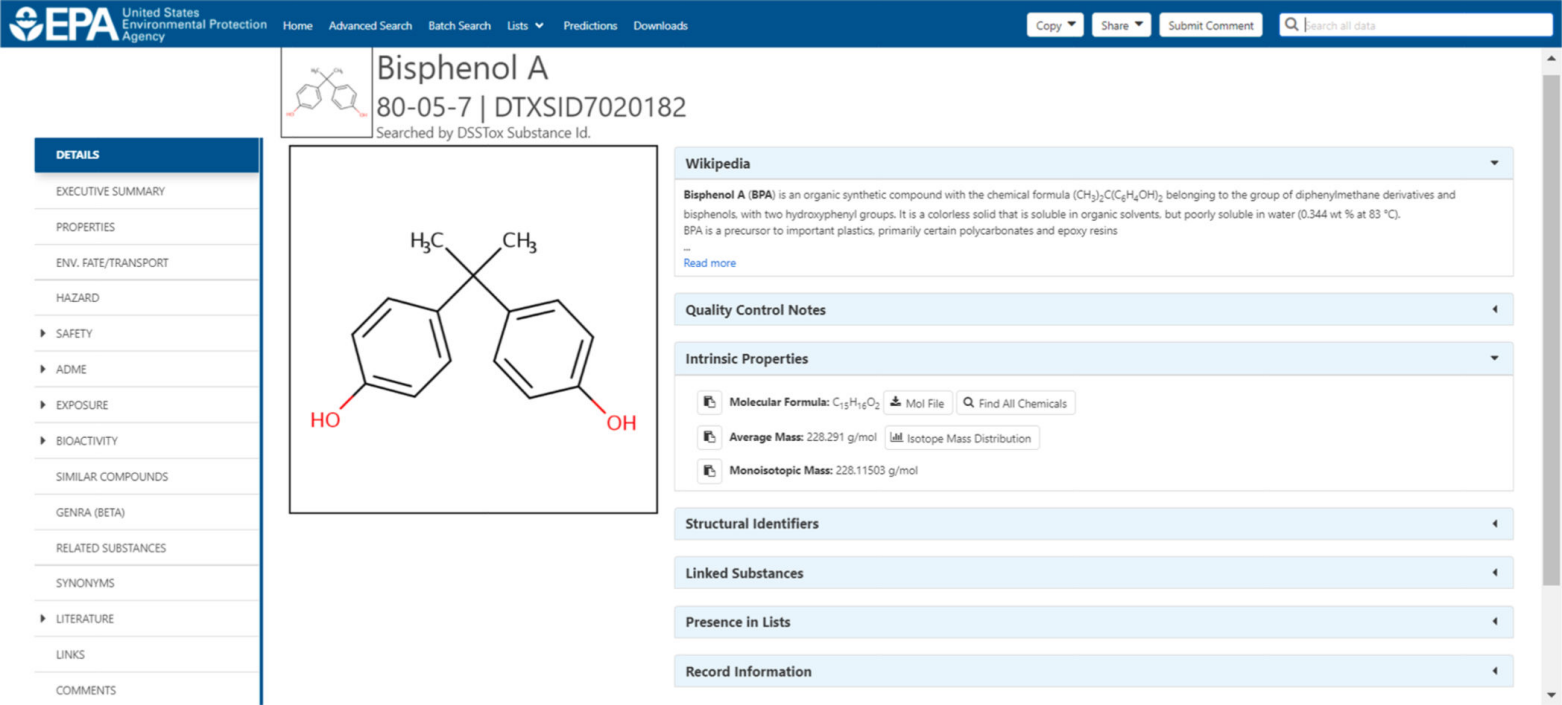
The results page for a search on the chemical name Bisphenol A. The subtabs on the left hand side of the results page navigate between different types of data.

Searching for a chemical on the Dashboard, based on an identifier such as a chemical name or CAS RN, will *generally* result in a single summary display of a chemical homepage. As an example, searching on the CAS RN 80–05–7 (for Bisphenol A) gives the homepage result shown in Fig. 1 (Chemicals, 2020).

**Fig. 2.**
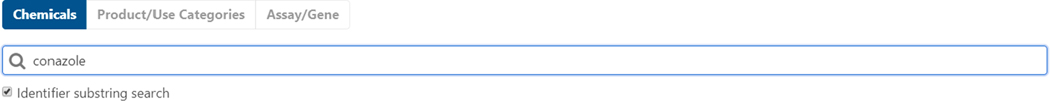
The substring selection box underneath the search box on the homepage will return all chemical substances containing that particular substring in either the preferred name or synonym table.

**Fig. 3.**
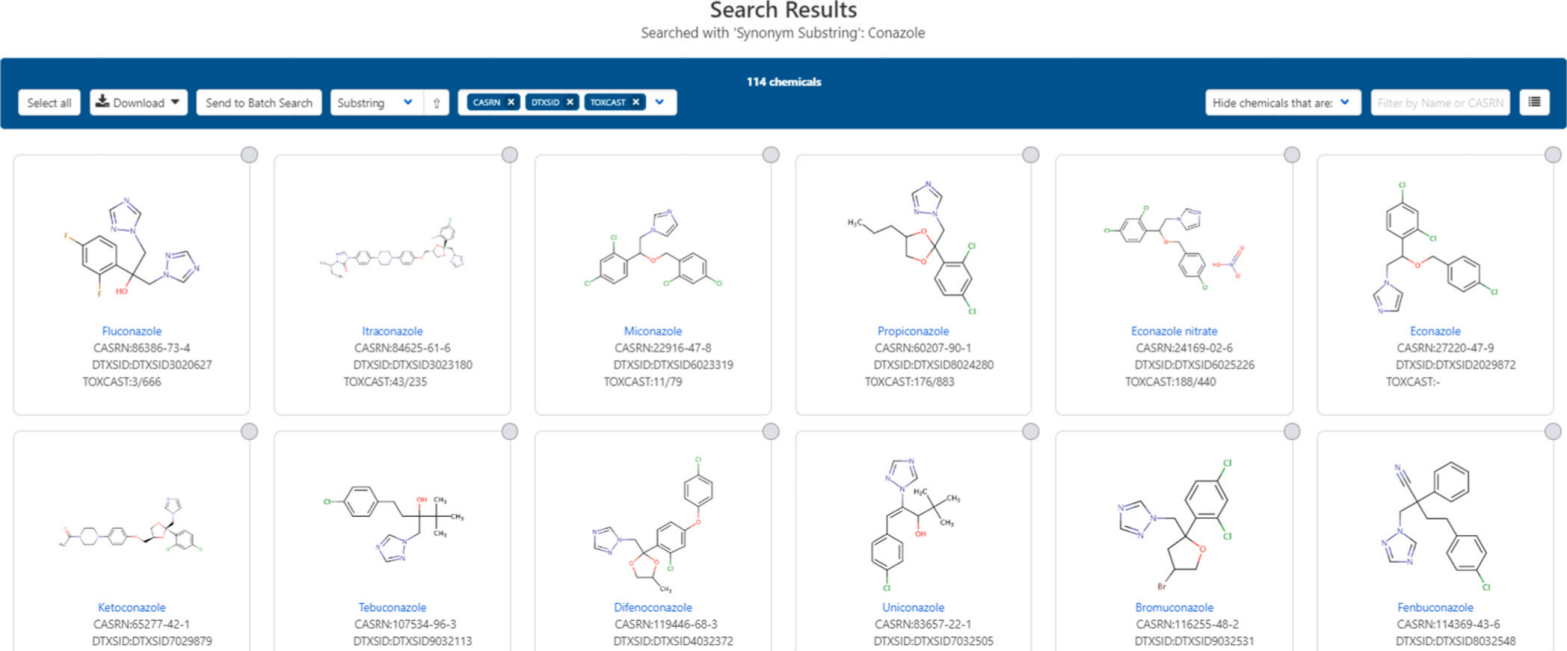
The results page from a search on the substring “conazole” returns over a hundred chemical substances. The resulting dataset can be downloaded in multiple formats (i.e., Excel, SDF, or tab-separated values). The dataset can be filtered to remove isotopically labeled chemicals and multi-component chemicals.

There are multiple sub-tabs on the left-hand side of a single chemical display where the user can expand and examine various types of data that should be evident from the title on the sub-tab (e.g., properties, synonyms, bioactivity, etc.). In contrast to the single result page shown in Fig. 1, a “multiple results” page displays a set of chemicals associated with a search and can result, for example, from a synonym substring search. For example, a search for the substring “conazole” through the home page using the “Identifier substring search” checkbox below the search box (See Fig. 2), yields multiple search results containing the name fragment “conazole” in either the preferred name field or the synonym table (see Fig. 3 and reference (Chemicals, 2020).

From the multiple results page the results set can be downloaded as a structure-data file (SDF) file (Dalby, 1992), an Excel file or tab-separated (TSV) variant. The file will be populated by default with the preferred name and CAS RN for each substance, as well as structure representations such as SMILES strings, InChI strings and InChI keys, formulae, masses, and other related metadata. It is also possible to include other data in the export clicking on the “Send to Batch Search” tab above the chemical images, which provides additional data export options (*vide infra*).

## 3. How do I use the Dashboard to find chemical synonyms?

Often a chemical listed on the Dashboard can be associated with a large number of synonyms, sometimes exceeding hundreds. The synonyms tab on the main chemical search results page contains a compiled list of both systematic and non-systematic names, trade names, trivial names, CAS RNs, Beilstein IDs and U.S. Food and Drug Administration (FDA) registry numbers. Synonyms listed on the Dashboard were auto-loaded from public websites (e.g., ChemIDPlus) added as a result of manually tagging new chemical lists into the Distributed Structure- Searchable Toxicity (DSSTox) chemical registration system (Grulke, 2019) by members of EPA/ORD’s chemical curation team, and generated using systematic nomenclature software. The data are displayed in the Dashboard using three font styles: *italicized* for Good Synonyms (indicating consensus across a series of public databases), **bold** for Valid Synonyms (manually curated by the team or algorithmically generated by systematic naming software), and normal font for Other Synonyms. The synonyms table can also include additional CAS RNs (deleted or alternate) that have been publicly associated with the substance, but not assigned by DSSTox curators as the “Active CAS RN”. There is only one Active CAS RN uniquely assigned to a DSSTox Substance, but there can be multiple deleted variants, even hundreds (see, e.g., (Chemicals and Dashboard, 2020).

## 4. How might the chemical synonyms located in the Dashboard be used to support human health assessment?

The variety of synonyms available on the Dashboard can be used to help construct literature search strategies for assessments. For example, the EPA Integrated Risk Information System (IRIS) (IRIS, 2020) and Provisional Peer-Reviewed Toxicity Value (PPRTV) (PPRTV, 2020) programs have recently begun to identify synonyms for inclusion in data search strategies based on identifying those indicated as “valid” or “good” from the Dashboard. Although there can be a large list of synonyms associated with a particular chemical, experience has shown that utilizing only the valid and good synonyms yields more reliable results. This was illustrated in recent work performing literature searches for azo dyes (publication in preparation). Currently, aggregating the preferred chemical name, CAS RN, and “valid” or “good” synonyms into a syntax used to search databases such as PubMed (Library, 2020), Web of Science (Web, 2020) or Scopus (Elsevier’s Scopus, 2020) is a manual process. In Table 1 a list of the different text strings used to query against two specific collections, PubMed and Web of Science, are shown.

## 5. The Dashboard is a “chemicals dashboard” but how do I find *in vitro* bioactivity data associated with the chemicals?

The ToxCast and Tox21 *in vitro* high-throughput chemical screening programs (Toxicity Forecasting: Advancing the Next Generation of Chemical Evaluation, 2020; Testing, 2020) were designed in response to the 2007 NRC report “Toxicity Testing in the 21st Century: A Vision and a Strategy” (Testing, 2007). The report called for the use of *in vitro* assays to ascertain the biological pathway-based effects of thousands of untested or data-poor chemicals. The goals of the ToxCast (Toxicity ForeCaster) program are to identify *in vitro* assays and responses relevant to *in vivo* phenotypic level toxicity and, from the resulting data, to develop predictive models to screen environmental chemicals that have little or no available *in vivo* toxicity data and prioritize them for further testing (Dix, 2007). The *in vitro* assays expose living cells, isolated proteins, or other biological molecules to chemicals, and measure qualitative and quantitative (concentration–response) changes in biological activity that may suggest the potential for toxic effects. Both ToxCast and Tox21 have been generating HTS bioactivity data for over a decade and the Dashboard provides access to both the summary views of the data as well as experimental concentration–response curves for individual chemicals (generated using models implemented in the ToxCast data pipelining tool: tcpl (Filer, 2016)).

**Fig. 4.**
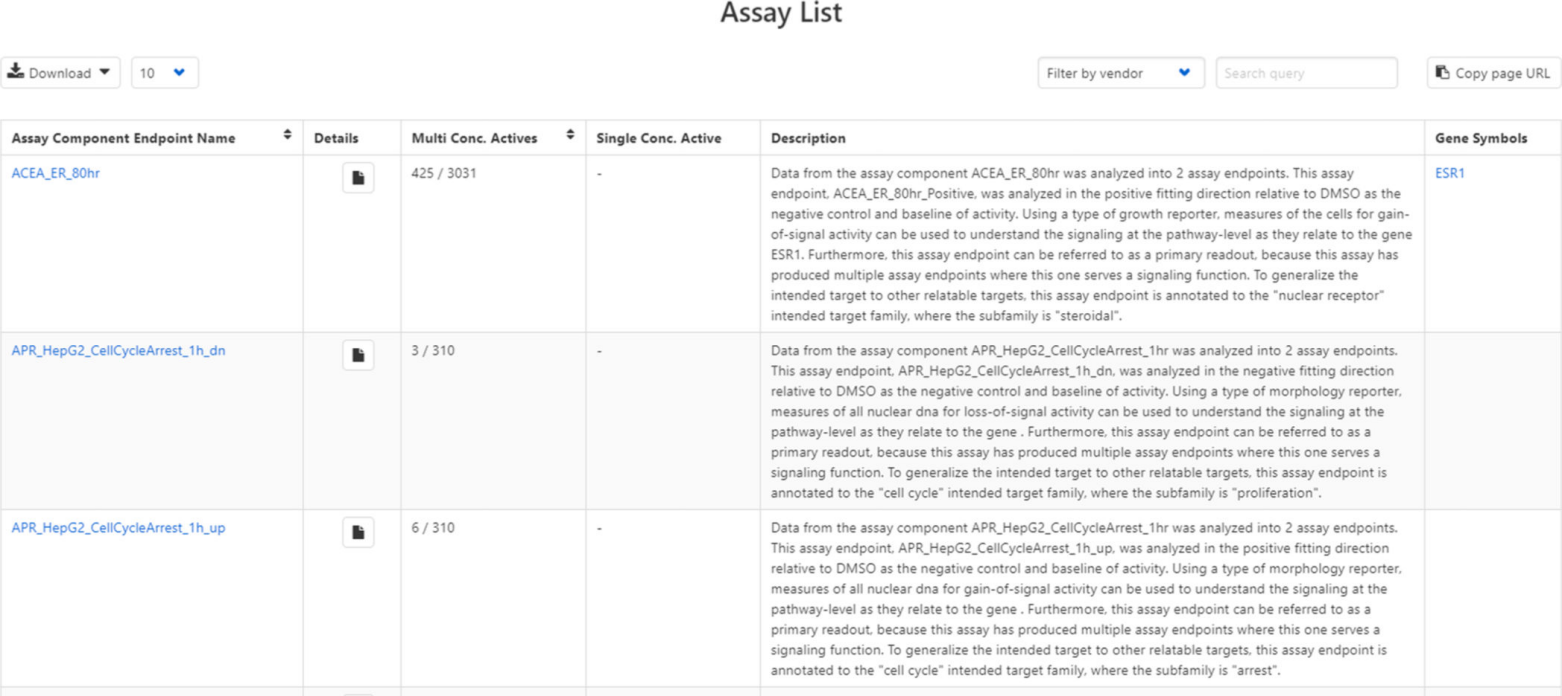
Sample view of the list of bioactivity assay endpoints available on the Dashboard (Chemicals, 2020). The table can be filtered using a search term on the top right-hand side (see below for an example) and the entire list can be downloaded into a file using the Download link in the top left-hand corner. Details regarding each of the assays can be found under a pop-up modal and includes links to articles regarding the assays, reagent details, and data processing details associated with tcpl (see below).

A list of all HTS assay endpoints that are available via the Dashboard is accessed through the top menu under Lists > Lists of Assays (see Fig. 4 and (Chemicals, 2020)).

**Fig. 5.**
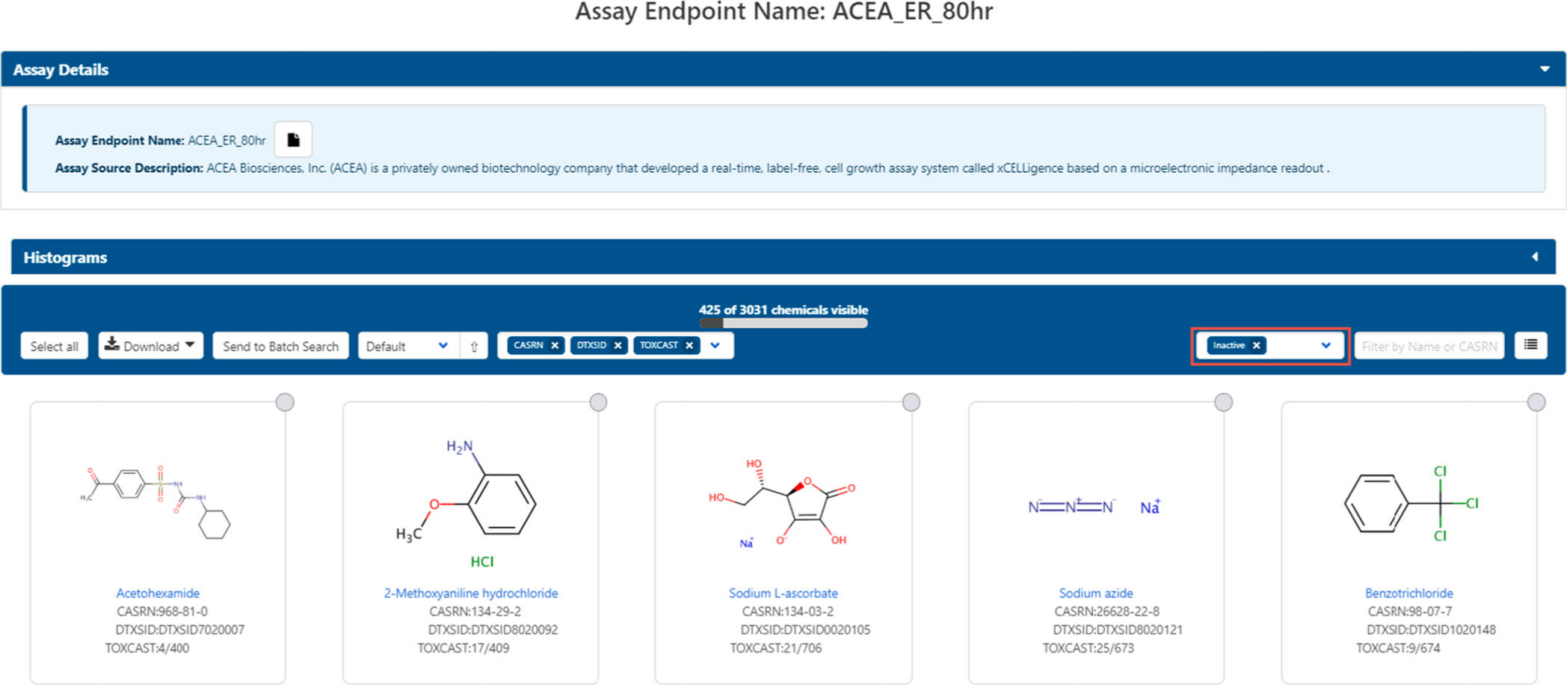
The list of chemicals associated with one of the assays: ACEA_ER_80hr (Chemicals, 2020) and Supplementary File 1) showing 425/3031 chemicals with active hitcalls. To view all chemicals, remove the Inactive filter (highlighted in the red block). The file can be downloaded as an Excel, SDF, or TSV file.

A total of 17 vendors (or laboratories conducting the testing) and 460 gene targets are currently annotated in association with the list of ToxCast and Tox21 assay endpoints, with assay endpoint list filtering enabled for either annotation. Selecting any of the specific assays will open an assay view of the chemicals that have been studied by that assay and can be filtered to display those with active or inactive hitcalls; each assay endpoint is assigned an assay-specific cutoff for a ‘positive hitcall’. For each chemical-assay combination, a hitcall (1/0, active/inactive, positive/negative) is defined, along with other parameters, including an efficacy value (Top) and potency value, called the AC50 or concentration at 50% of maximal activity. Note that in some cases an AC50 value is given even when the hitcall = 0. In this case the hitcall overrules the AC50. Some examples of the efficacy/Top values might be 20% change compared to control, or 3-fold baseline median absolute deviation, etc. In addition, for some assay endpoints, the efficacy cutoff for a positive hitcall is statistically defined, whereas for others there may be some known magnitude of biological change that is relevant. In general, a positive hitcall is defined as a biological perturbation having a maximum median response that exceeds the cutoff defined for the assay and having data that can be curve-fit. For example, the ACEA_ER_80hr assay chemical list (Chemicals, 2020) as initially displayed when opening the chemical list, displays the 425 (out of 3031 studied) chemicals with active hitcalls for this specific estrogen receptor assay. The entire chemical dataset can be displayed by deleting the “Inactive” filter in the header above the chemical list (and highlighted in Fig. 5). The dataset displayed in this multiple chemical results view can be downloaded to the desktop for viewing in any of the usual formats described above.

Although the abbreviated name of a given ToxCast/Tox21 assay endpoint might appear rather obscure or non-descriptive, such as ‘ACEA_ER’, the details of the ACEA_ER_80hr assay, a cell-based, single-readout platform that uses the T47D human breast cell line with measurements taken at 80 h after chemical dosing, is described in detail in the “assay modal” (i.e., by clicking on the page icon located next to the assay name). This pop-up contains explanatory text regarding how the assay is measured, citations generally describing the development, validation and applications of the assay, details regarding how data from the assay are pipelined through the ToxCast data processing pipeline (*vide infra*), reagents used in the assay, and other relevant data..

The ToxCast program has been conducted in multiple phases and, to date, has studied greater than 4500 chemicals (US EPA ToxCast Chemicals webpage, 2020) in one or more assays, with the full chemical list available via the Dashboard (Chemicals, 2020). An overview of the testing phases and different types of chemicals that have been studied is provided online (US EPA ToxCast Data Generation: Chemical Lists, 2020) and discussed in detail in a recent paper (Richard, 2016). The Tox21 program, also conducted in phases (Richard, 2020), has screened more than 8000 chemicals, a list of which is also available via the Dashboard (Chemicals, 2020). A number of papers regarding how to interpret the results associated with ToxCast and Tox21 data have been published (Filer, 2016; Judson, 2010; Judson, 2016). This section has summarized how to access overviews of those bioactivity assays that have screened more than a few thousand chemicals. However, a deeper, interpretive analysis of the data for each chemical is reviewed in the next section. The bioactivity data associated with a single chemical requires navigation to a chemical with available data (out of nearly 9000 chemicals in total screened in either Tox21 or ToxCast assays) and then perusing the data through the bioactivity data tab for that chemical.

## 6. I can find the high-throughput screening (HTS) results for a chemical, but how do I interpret them?

**Fig. 6.**
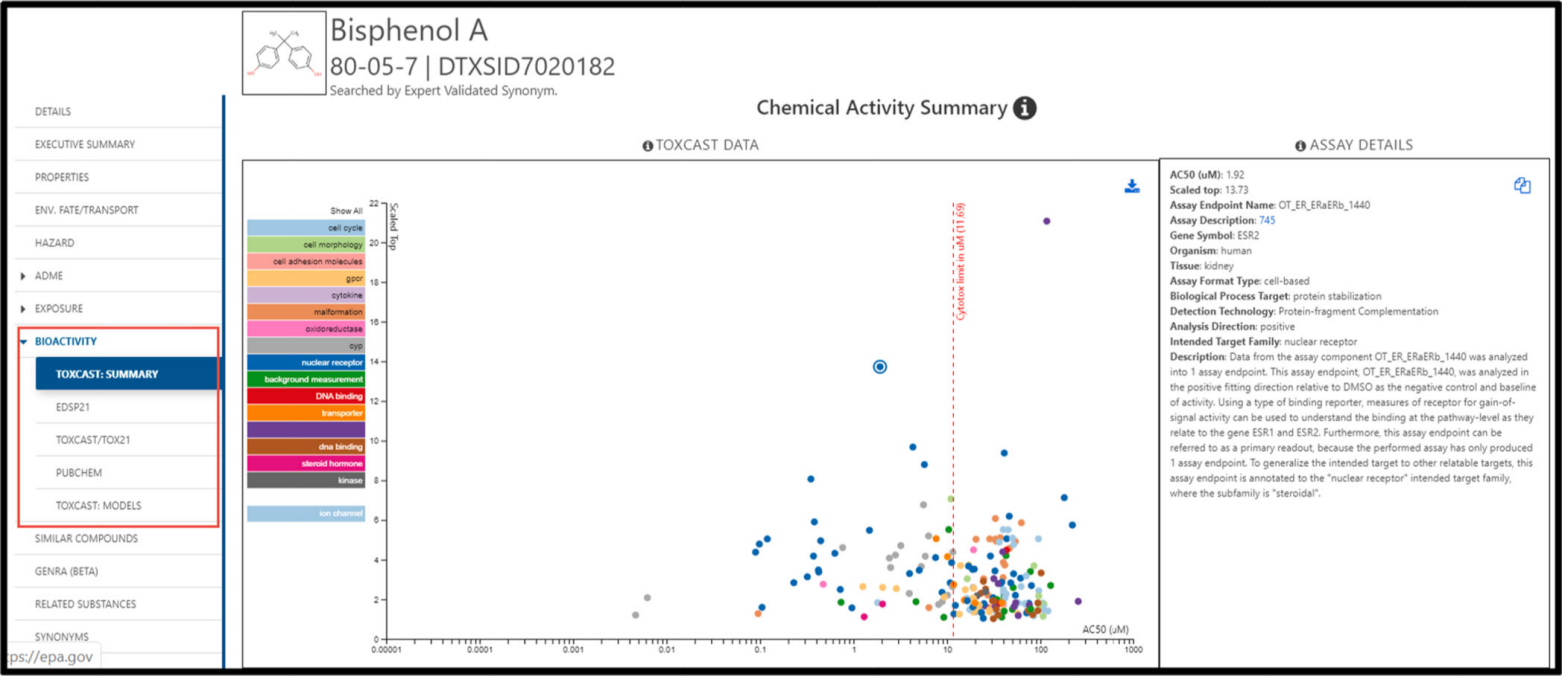
The ToxCast summary view showing active hitcalls for all assays performed on Bisphenol A. The table under the summary plot contains all of the measured values associated with the array of assays.

The previous question provided an overview of how to query, surface, and display the bioactivity data available for ~ 4750 chemicals associated with the ToxCast program (Chemicals, 2020) and more than 8000 chemicals for the Tox21 program (Chemicals, 2020). It should be noted that bioactivity data are available for only a small fraction of the ~900,000 chemicals represented on the Dashboard. Hence, the bioactivity tab may commonly be greyed out when no data are available for a given chemical of interest. The assays themselves are also segregated into two specific cluster sets associated with the ToxCast/Tox21 programs as a whole (i.e., all assays) and the subset of the assays assigned as relevant to the Endocrine Disruption Screening Program for the 21st Century (EDSP21) that was established to prioritize and screen chemicals to determine their estrogen, androgen or thyroid bioactivity (US EPA Endocrine Disruptor Screening Program (EDSP) in the 21st Century, 2020). The list of chemicals of interest to the EDSP21 study, the so-called EDSP universe, is available online (Chemicals, 2020). This segregation of data into two separate clusters of assays is obvious when viewing the data under the Bioactivity sub-tab as shown in Fig. 6. There are five individual sub-tabs under the bioactivity tab that represent: (i) the summary data visualized with a set of color-coded targets, each point on the graph displaying associated assay details on the right hand side of the plot. Note that a table of the data in the graphical plot is displayed below and can be filtered and downloaded to the desktop in the usual Excel or TSV formats; (ii) the EDSP21 subset of assays annotated as associated with the estrogen receptor (ER), the androgen receptor (AR), thyroid and steroidogenesis; (iii) the entire set of all assays run under the ToxCast and Tox21 programs and segregated in the interface according to the individual assay vendor or laboratory conducting the measurements; (iv) a search of the PubChem database displaying any bioassay data for the chemical refreshed in real time and listed in a PubChem widget (PubChem: Substance Bioactivities Table Widget Example, 2020) embedded in the Dashboard; (v) ToxCast endpoint prediction model data associated with a series of derived computational toxicology models including pathway models (Browne, 2015; Kleinstreuer, 2016), the CERAPP model (Collaborative Estrogen Receptor Activity Prediction Project) (Mansouri, 2016) and the CoMPARA model (Collaborative Modeling Project for Androgen Receptor Activity) (Mansouri, 2020).

The ToxCast Users’ Manual is a valuable resource for understanding the details of how to interrogate the available bioactivity data (US EPA ToxCast Owner’s Manual - Guidance for Exploring Data webpage, 2020). In brief, the color-coded panel down the left side of the scatter plot in Fig. 6 identifies the biological grouping or category for various plotted points (e.g., DNA binding; cytochrome P450 activity [cyp]; etc.). It should be noted that all active hitcalls are visualized in the plot; clicking on any one of the category panels on the left will hide/turn off the plotted points for that category. The plotted points represent the AC50 (in uM) for a given activity, where AC50 = the concentration at which half the maximal response for the specific assay was observed. Clicking any one of the points will display the assay details to the right of the plot. Lastly, the vertical dashed red line in the scatter plot represents the *in vitro* cytotoxic threshold concentration for a chemical, commonly referred to as the “cytotoxic burst” point (Judson, 2016). As such, points plotted to the right of this burst concentration represent activities that are concomitant with or potentially a result of cell stress or cytotoxicity, and hence not indicative of direct or specific activity of the chemical against the intended target of the assay. Further descriptive details of the ToxCast platform(s) and data visualization and interpretation are found in the Users’ Manual (see link above). The manner by which individual concentration–response curves can be interrogated is dealt with in the next question.

## 7. How do I extract concentration–response data for specific ToxCast assays?

**Fig. 7.**
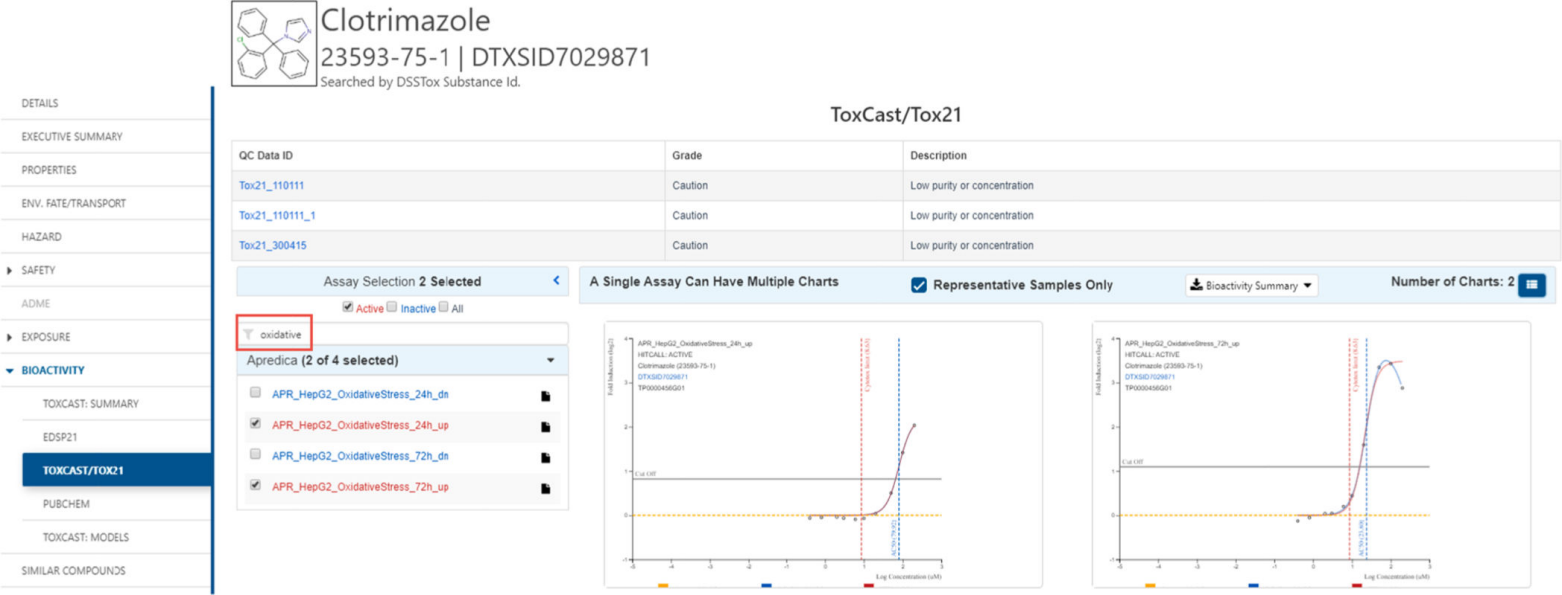
Selection of the ToxCast/Tox21 set of assays for the chemical will list all associated assays. Checking the Actives red checkbox will select all assays with active hit calls. Adding the filter “oxidative” filters all assays with the term in the assay name. (For interpretation of the references to color in this figure legend, the reader is referred to the web version of this article.)

Dose-response data for a specific chemical, and for a specific assay, can be extracted from the Dashboard for further analysis and re-fitted if required. For example, let us assume the investigation of a specific chemical, such as Clotrimazole, for potential oxidative stress is of interest (Chemicals, 2020). Oxidative stress associated with Clotrimazole can be examined across all ToxCast/Tox21 assays by searching for all active hitcalls (selecting the Active Hits) and adding the text string “oxidative” to view the related concentration–response curves as shown in Fig. 7 (Chemicals, 2020). The parameters associated with the individual fitted curves can be downloaded (as Excel or TSV) by selecting the Bioactivity Summary download file.

**Fig. 8.**
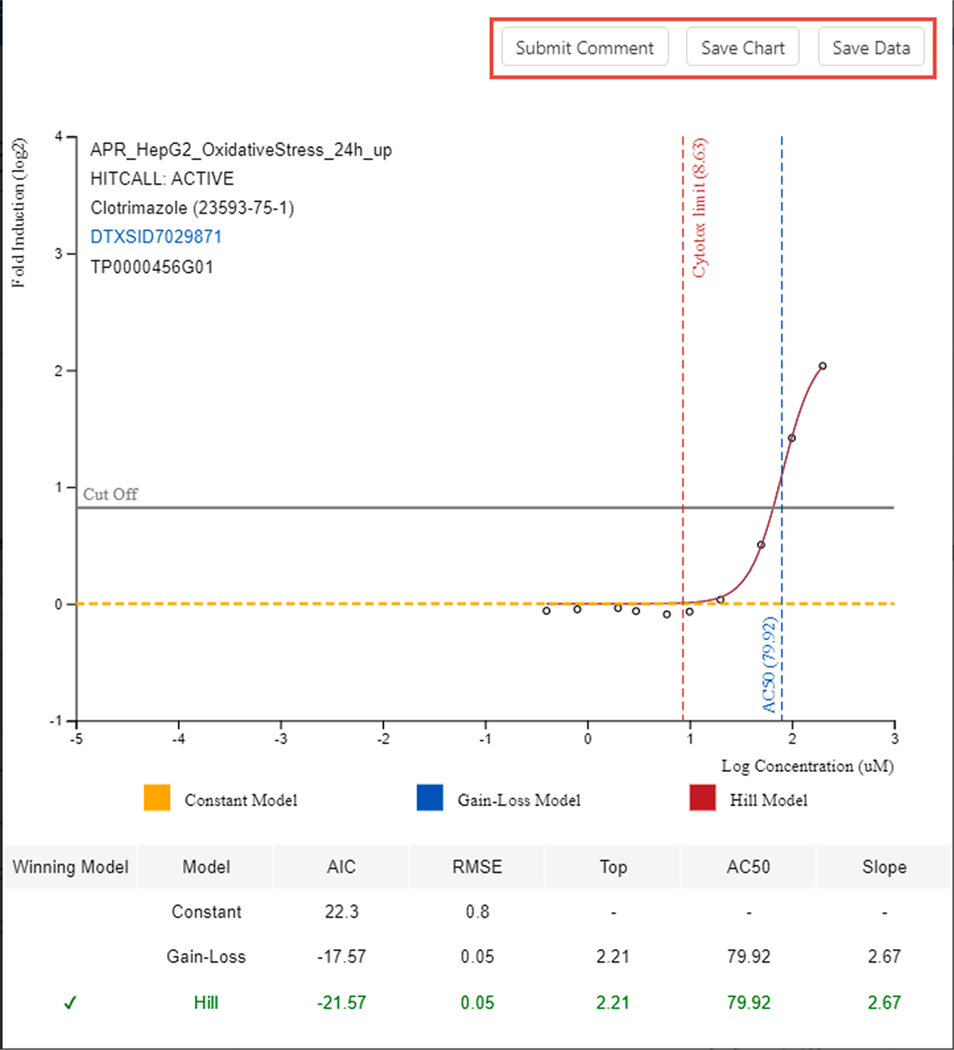
An individual concentration–response curve can be viewed, and the image and associated data points can be downloaded.

Each of the individual concentration–response curves can also be downloaded, either as the image or as a table of individual points (see Fig. 8).

**Fig. 9.**
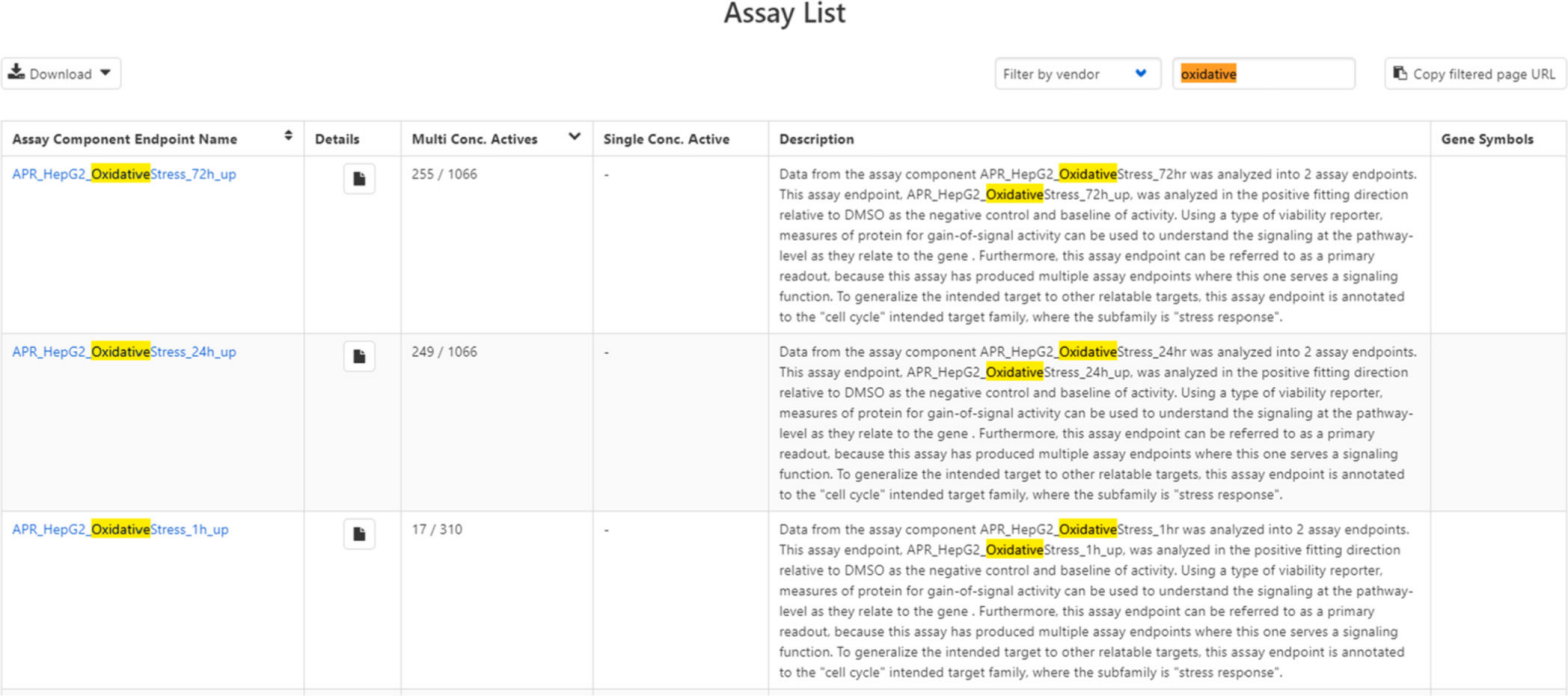
Filtering through the List of Assays page based on an input filter-string of “oxidative”.

For reviewing a collection of chemicals that may exhibit oxidative stress, a user would browse the list of assays (Chemicals, 2020) and filter based on the term oxidative (see Fig. 9).

## 8. How do I use the Dashboard to conduct a read-across analysis?

Read-across, in general, is the comparative evaluation of chemicals across similarity contexts such as structure, physicochemical properties, toxicokinetics, and/or bioactivity/toxicity. The objective is to inform data-poor chemical(s) by leveraging hazard and dose–response information from one or more “similar” data-rich analogue chemicals. A read-across module is embedded into the Dashboard and can be applied to a large majority of the chemicals in the database. The Generalized Read-Across (GenRA) workflow (Shah, 2016; Helman, 2019) is available under a sub-tab in the left menu panel of the main chemical result page. The module identifies source analogues and makes predictions of *in vivo* toxicity effects for a data-poor target substance.

GenRA allows users to identify analogues in different ways; by default, chemical fingerprints are used (RDkit’s Morgan and Torsion fingerprints as well as ToxPrint chemotypes (Yang, 2015) though ToxCast and Tox21 hit call data expressed as fingerprints can also be used. Analogues returned are automatically pre-filtered on the basis of availability of *in vivo* data for over 1000 substances within ToxRef v1 (Martin, 2009). ToxRef v1 data have been binarized to reflect toxicity effects such as kidney effects, body weight changes within 10 different study types (e.g., subacute, subchronic, chronic). Different study types and the effects within those studies can be predicted: e.g. chronic_liver is annotated as CHR_liver. Negative results which are reported assume that if a particular guideline study was conducted but effects were not reported then a chemical would be negative for that particular effect for that type of guideline study. Positive results, however, represent the minimum dose at which toxicity effects are observed in a study. ToxPrint chemotypes are structural fragments that can encode various properties (i.e., physicochemical, atomic, and electronic), in addition to the form of a particular chemical substructure. These chemotypes also can be independently associated with biological properties and modes of action in toxicity pathways.

**Fig. 10.**
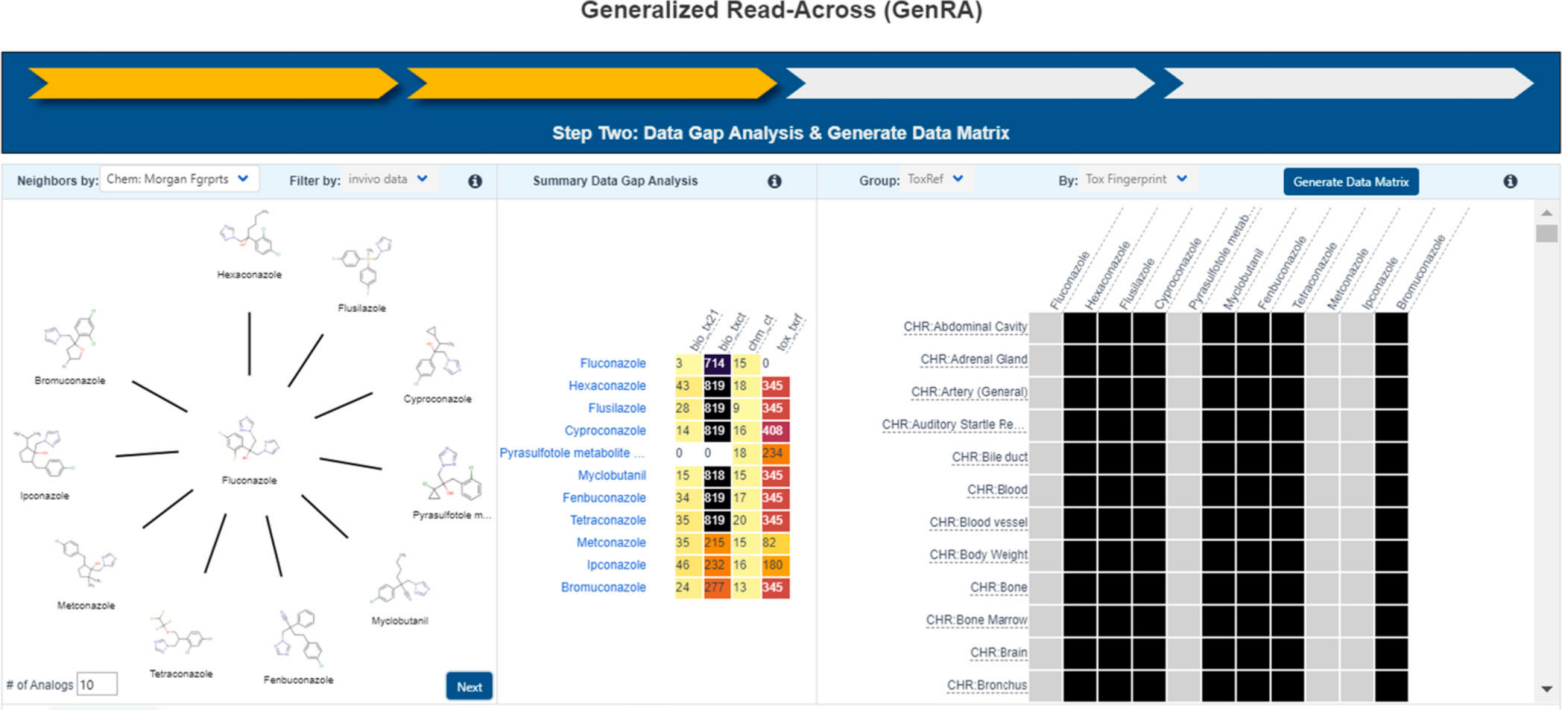
GenRA follows a multi-step workflow as displayed by the tracker above the various blocks of the workflow. As indicated for the read-across for fluconazole, at the center of the clock-face of similar structures, analogues have been selected using “Chemical Fingerprints”. The second block indicates data availability for each of the analogues in terms of the various data-types: Tox21, ToxCast, and ToxRef, with the numbers and the colors indicating the amount of data. The third block shows the availability of data for each of the toxicity endpoints as a binary display (black indicates available data).

GenRA’s workflow (Helman, 2019) is meant to mimic the workflow that a read-across expert would tend to follow. However, it should be noted that the scope and depth of this workflow-based assessment is currently limited only to chemical structure similarity as the main similarity context. The progress through the various stages of the workflow are indicated by progress in the tracker displayed at the top of the interface (i.e., the yellow arrows). The user first selects from the pulldown the type of fingerprints that they wish to use for the read-across. The selection of the relevant analogue(s) is represented in block 1 of the workflow described previously and shown in Fig. 10. The second block indicates data availability for each of the analogues in terms of the various data-types - Tox21, ToxCast and ToxRef - with the numbers and colors indicating the amount of data. The third block shows the availability of data for each of the toxicity endpoints as a binary display (black indicates available data). The user selects “Generate Data Matrix” to produce the initial read-across matrix that the user can interrogate (see Fig. 10).

**Fig. 11.**
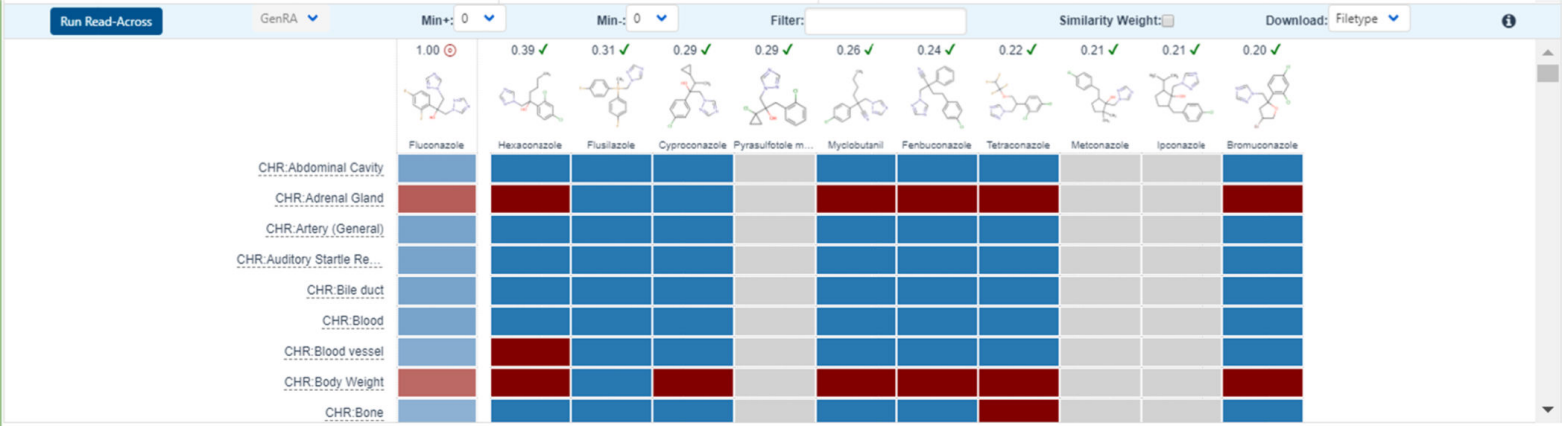
The final stage of the GenRA workflow is running the Read-Across function. The result is a binary hit-call displayed (in the first column) with red indicating active and blue inactive against a specific toxicity endpoint. (For interpretation of the references to color in this figure legend, the reader is referred to the web version of this article.)

The data are visualized in a data matrix (see Fig. 11) to evaluate the consistency and concordance of the available experimental data for those analogues before making a GenRA prediction.

The resulting predictions can then be exported into a tab-separated value (TSV) or Excel file for additional review and analysis (e.g., doses of analogues associated with production of toxic effects, see supplementary information file S.I.2). The predictions are presented as binary outcomes reflecting the presence or absence of toxicity, as well as providing quantitative measures of uncertainty. For more information on GenRA, the reader is referred to the article describing the workflow in detail (Helman, 2019), along with an accompanying user manual for GenRA (GENRA generalized read-across manual, 2020) and an overview video showing step-by-step walk-through of the module (Patlewicz and GenRA, 2017).

## 9. What sorts of data are provided in the Hazard” subtab module, and how do I assess their appropriateness for use in a risk assessment context?

The data available under the Hazard tab are associated with the ToxValDB database, which was designed to store a wide range of public toxicity information in a less restricted, more summarized form than another EPA database, ToxRefDB (Watford, 2019). ToxRefDB consists of data extracted from thousands of animal studies and, for the latest release version, contains data for over 1100 chemicals (Chemicals and Dashboard, 2020), the majority pesticide guideline studies, and is one of the largest *in vivo* toxicity databases publicly available. However, the information requirements for transparency, study rigor, and results reporting in the ToxRefDB database prevents the integration of less detailed data from many other sources. ToxValDB, in contrast, spans a much larger domain of chemicals and collates publicly available toxicity dose–effect related summary values typically used in risk assessments. These include Point-of-Departure (POD) data collected from data sources within ACToR (Judson, 2008) and ToxRefDB, and no-observed and lowest-observed (adverse) effect level (NOEL, NOAEL, LOEL, LOAEL) data extracted from repeated dose toxicity studies submitted under REACH (ECHA REACH, 2020). Also included are non-cancer reference dose (oral route) and concentration (inhalation route) values (RfDs and RfCs) from IRIS and PPRTV databases. Acute toxicity information was extracted from a number of different sources, including the OECD eChemPortal (OECD eChemPortal: Global Portal to Information on Chemical Substances, 2020), the European Chemicals Agency (Agency, 2020), NLM (National Library of Medicine) HSDB (Hazardous Substances Data Bank) (Bank, 2020); ChemIDplus (Website, 2020) via EPA’s TEST (Tool, 2020), the EU JRC (Joint Research Centre) AcutoxBase (Kinsner-Ovaskainen, 2009), and the EU COSMOS project (COSMOS: Integrated In Silico Models for the prediction of Human Repeated Dose Toxicity of COSMetics to Optimize Safety website, 2020). In the context of conducting assessments, ToxValDB can be a very valuable resource to identify grey literature associated with regulatory activities (e.g., unpublished industry-sponsored toxicity studies) that is not published in open domain assessment repositories or journal articles. However, it is important to note that most ToxValDB entries have not yet undergone quality control to ensure accuracy or completeness. Also, ToxValDB data may not include recent studies, especially since data releases are currently on a six-month cycle. Further, many chemical assessments were completed over 20 years ago. Thus, it is best used as an assist and complement to manual approaches to identify updated public domain studies and/or grey literature.

An important aspect of toxicity effects, the POD, and/or toxicity value data provided in ToxValDB, particularly when mined from assessment reports and databases, is that the primary source would be the principal study or studies on which the information was derived rather than the corresponding published assessment (which might be considered the secondary source). For example, a given hazard effect, associated POD, and resulting toxicity value would indeed come from an assessment report or document; however, the originating data comes from a published or grey literature human epidemiological or experimental animal study. Thus, in practice, it might be ideal to cite or refer to both the assessment report and the originating principal study on which the hazard identification and dose–response assessment was based.

At the time of this writing, the ToxValDB data collection (version 5, (Chemicals and Dashboard, 2020) includes data for ~ 56,000 chemicals on the dashboard with ongoing expansion of both the number of chemicals and the associated data. Ongoing ToxValDB data updates, which are on a more frequent basis to the Dashboard release, are available via FTP download (FTP site for accessing ongoing updates to ToxValDB data, 2020).

## 10. What types of physical chemical measurements and predicted properties are available in the Dashboard and what value should I choose in cases where there are multiple predicted values presented for the same entity (e.g., predicted average, mean, range)?

**Fig. 12.**
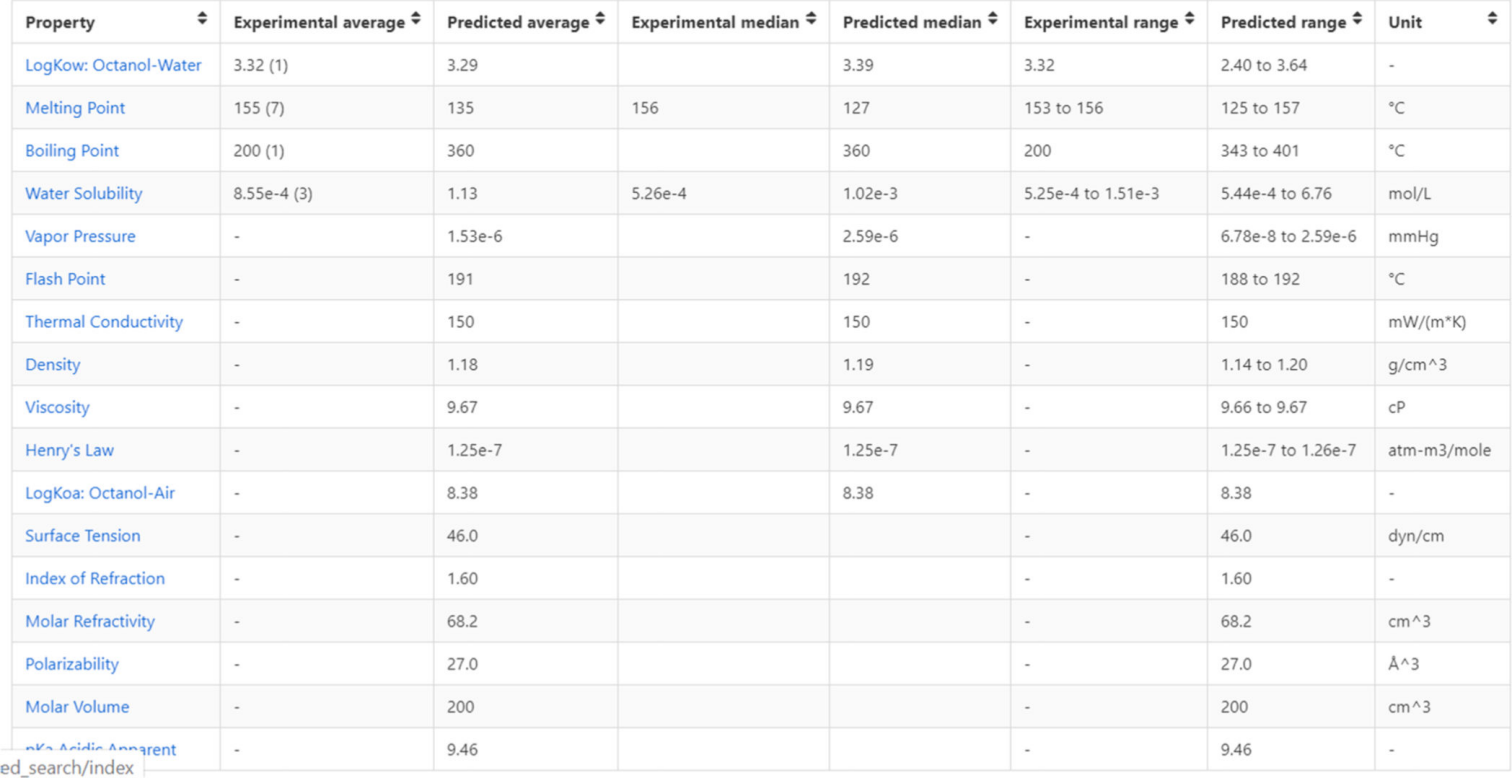
The summary table of both experimental and predicted physicochemical properties associated with Bisphenol A. Clicking on any of the blue hyperlinks for the property will open up a detail page for that property. The table includes averages, medians and ranges for both the experimental and predicted values. (For interpretation of the references to color in this figure legend, the reader is referred to the web version of this article.)

The Dashboard hosts both measured and predicted physicochemical data from many sources, including curated datasets associated with predictive models (Mansouri, 2016), online databases, and the peer-reviewed literature. Experimental data from provenanced data sources, such as peer-reviewed articles, with direct links to the article if possible, are generally preferred over modeled data but data are generally not available for all endpoints of interest and for all chemicals. Tens of thousands of data points for different endpoints have been extracted and mapped to the relevant chemicals, but of the ~900,000 substances on the Dashboard experimental data are only available for a fraction of the collection, e.g. ~15,000 logKow values and ~1,000 bioconcentration values. The property endpoints available for a chemical are represented in Fig. 12 for Bisphenol A (Chemicals, 2020). Notice that the table shows experimental data for only four of the properties, but predicted data are available for all of the endpoints.

**Fig. 13.**
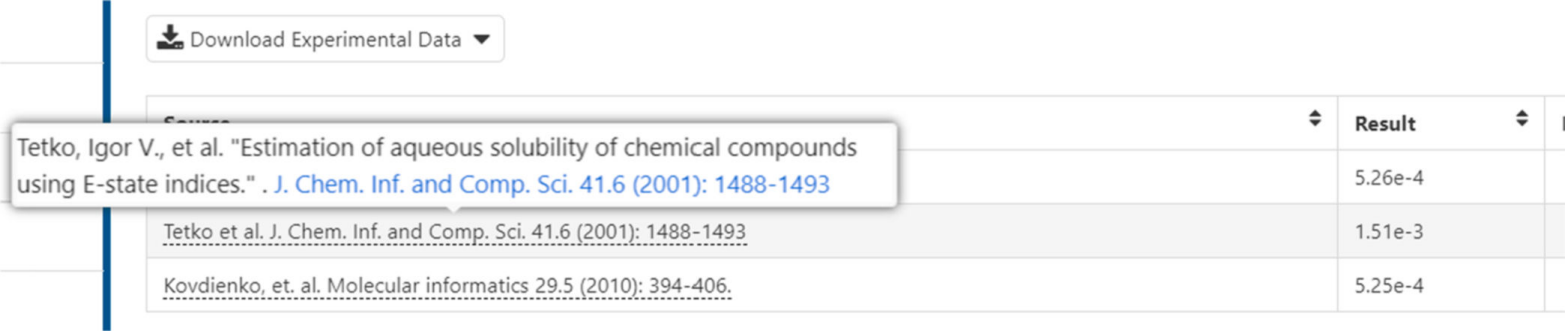
Provenance of the data back to the original source is provided where possible in the on-hover. As shown, the publication is hyperlinked using the article DOI (digital object identifier) to ensure no link decay.

In the first column of the table, blue hyperlinks for the individual properties are listed and clicking on one of the links will open up a detail page for that property listing the individual experimental values and on-hover information (see Fig. 13).

**Fig. 14.**
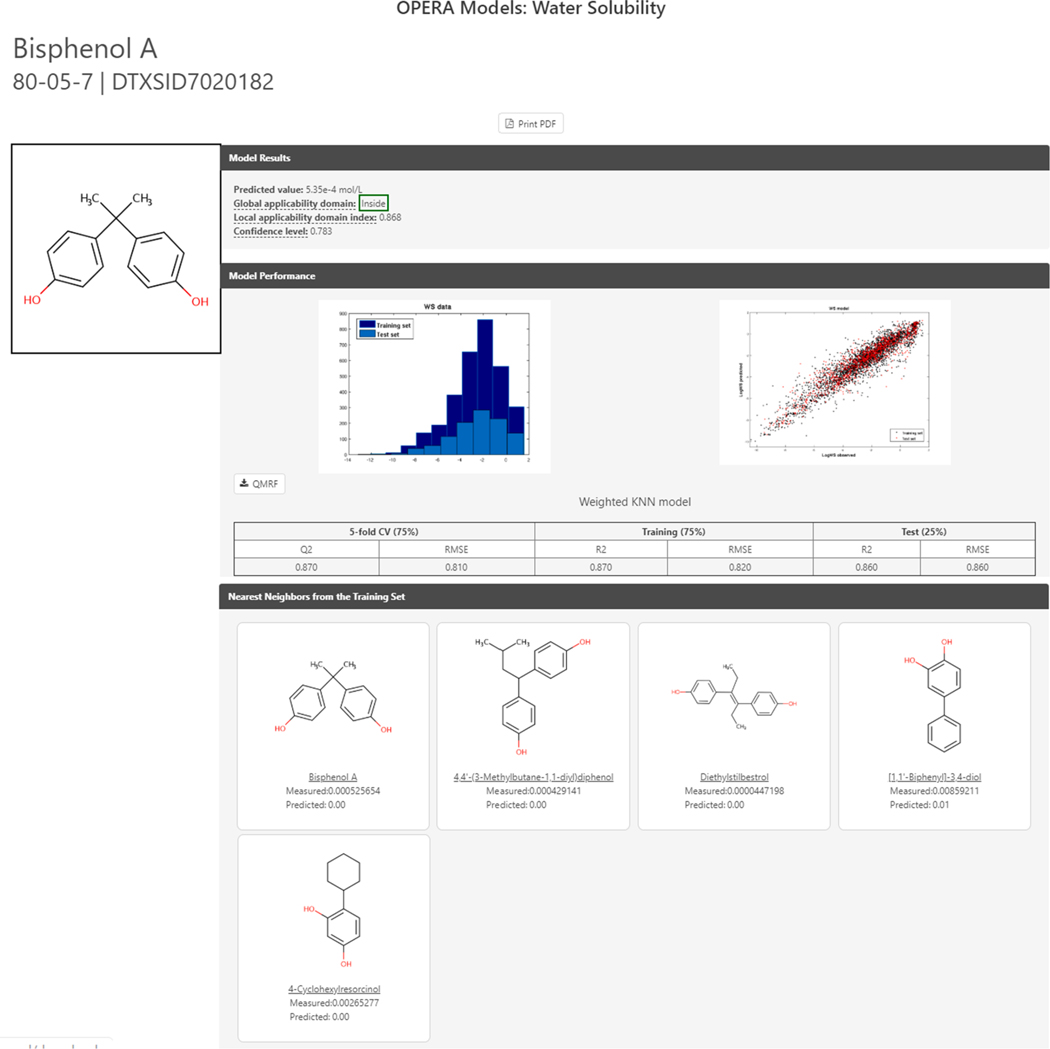
The OPERA calculation report for the predicted water solubility of Bisphenol A. The global performance of the model is displayed together with the applicability domain, and numeric reliability assessments between 0 and 1. It also provides up to five nearest neighbors from the training set (where available), with their experimental and predicted values as an additional reliability assessment for the user.

As well as listing experimental data for a given chemical, multiple predicted values are listed. The list of prediction sources depends on the individual property of interest as not all algorithms cover all properties. Prediction algorithms include EPI Suite (EPI Suite™-Estimation Program Interface, 2020), ACD/Labs (Development, 2020), TEST (Tool, 2020) and OPERA (Mansouri, 2018; Mansouri, 2019). The most complete set of predicted values, especially in terms of transparency regarding how the models are built and how they perform, are available for the OPERA models as these include links to QMRF reports (QSAR model report format), e.g., the OPERA Water Solubility QMRF report (Water Solubility QSAR Model Report Format, 2020) and detailed calculation reports (e.g., the OPERA water solubility prediction report (Water Solubility OPERA prediction report, 2020) as shown in Fig. 14. These reports have been created with the purpose of facilitating the use of such QSAR models for risk assessment and regulatory purposes while providing the results of predictions which are underpinned in the OECD guidance document on the validation Principles of QSAR models ([68]). Calculation reports are also available for the TEST predictions. Such detailed information is not available for either the EPI Suite or ACD/Labs predictions. Similar tabulated views of experimental and predicted data are available under a separate subtab for Environmental Fate and Transport properties, such as bioconcentration and bioaccumulation factors, again with QMRF details for OPERA and detailed calculation reports for TEST and OPERA models.

Concerning which value should be selected from the properties or environmental fate and transport sub-tabs, a user is recommended to review the data for a particular endpoint. If there are multiple experimental data points available, it is recommended that the average, median and range may all be useful to include with the reference URL for the data. If there are no experimental data available, then the predicted data can be consulted. Multiple prediction results are listed to indicate our agnostic approach to models. It is recommended that a user interrogate the calculation report to understand the performance statistics for that prediction. Consider the OPERA prediction report for water solubility for BPA (Water Solubility OPERA prediction report, 2020) which shows the global performance of the models, the applicability domain, and the reliability assessment. It also provides up to five nearest neighbors from the training set (where available), with their experimental and predicted values, as an additional reliability assessment for the user.

It should be noted that data may change with each release as more data are added and the models may be rebuilt with resulting changes in the prediction results.

## 11. What kind of analyses can I perform using the similar compounds and related substances tabs and how do these differ?

**Fig. 15.**
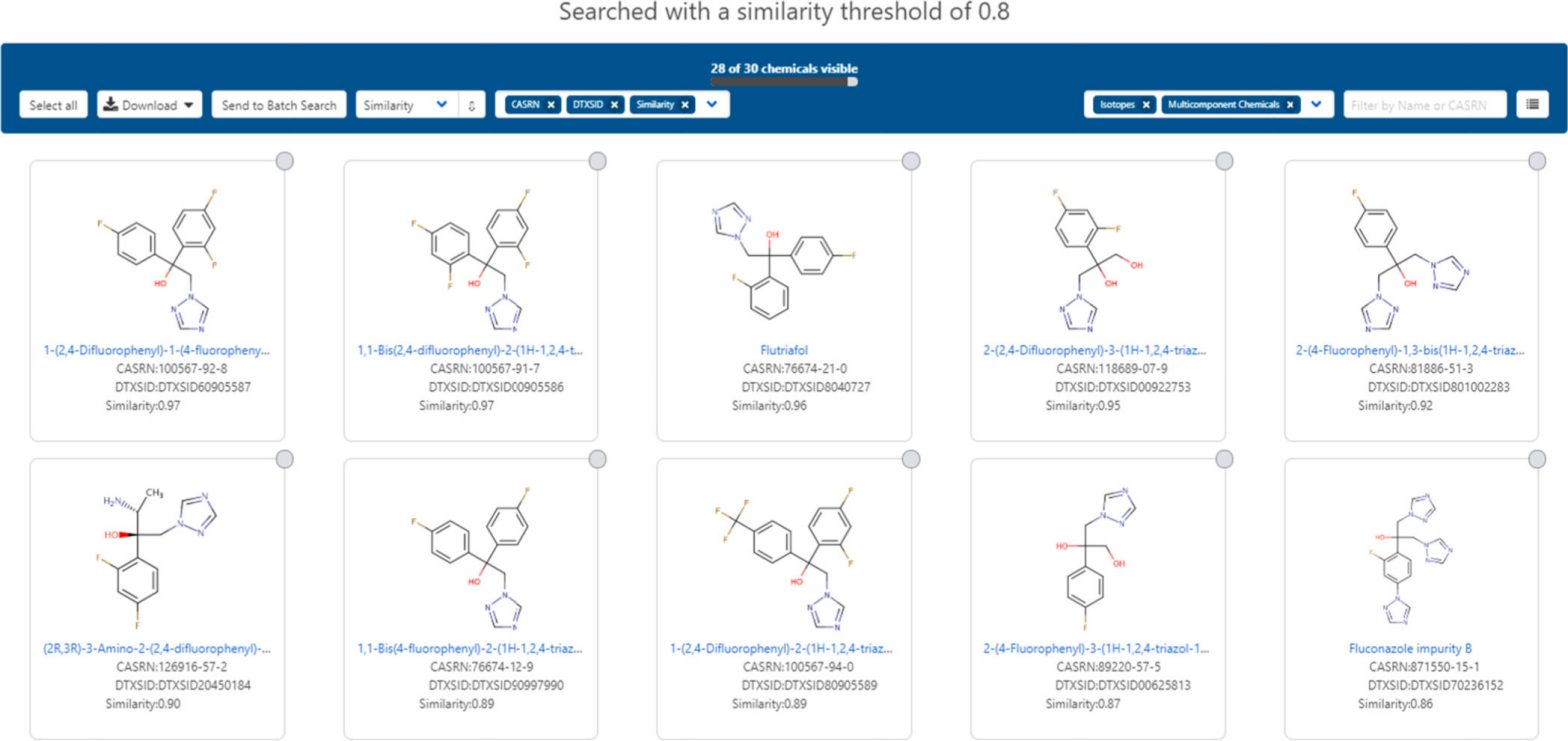
Similarity search results for Fluconazole based on a Tanimoto score of greater than 0.8.

The similar compounds tab represents the search results for a Tanimoto-based similarity search (Bajusz et al., 2015) and displays chemicals with a structural match factor above 0.8. For example, for fluconazole, 30 chemicals are displayed (with 2 isotopically labeled compounds not shown) (Chemicals and Dashboard, 2020) as shown in Fig. 15. These similar compounds can be used in a manual read-across process where those with replete hazard and dose–response data can potentially be leveraged to inform a data-poor target chemical, or, interrogated for other representative data that can be used for comparison purposes in an assessment.

The related substances tab contains chemicals that are related to the query chemical through some form of mapped relationship in the underlying database. Such relationships can include transformation products (e.g., for chlorothalonil (Chemicals, 2020) or as members of a chemical family (e.g., polychlorinated biphenyls, (Chemicals, 2020). The ability to retrieve and examine related substances can be of significant value for risk assessment purposes. For example, human metabolites or environmental degradants associated with a chemical may have experimental (or predicted) toxicities that should be taken into account.

## 12. How can I use Dashboard “Literature” subtab tools, such as the Abstract Sifter module, to text mine the published literature to aid in systematic reviews?

The Abstract Sifter module built into the Dashboard performs real time searches against the PubMed database (Library, 2020), containing 30 million abstracts at the time of writing. The Sifter uses the CAS RN and Preferred Name associated with a chemical, together with a set of pre-defined queries, to perform the search. The Abstract Sifter returns the number of occurrences of each term found in the title and abstract combined. The web-based spreadsheet’s citation counts can be sorted, and the module provides flexibility in further filtering with the option to create or enter more granular keyword search terms. The abstracts meeting the evolving search string criteria can be further filtered and elevated to the top of the heat map visualization as shown in Fig. 16.

The sifter is also available as a Microsoft Excel-based application and a link to download the application is shown on the webpage (Baker et al., 2017).

## 13. How do I extract available *in vitro* toxicokinetic data (ADME) from the Dashboard?

**Fig. 17.**
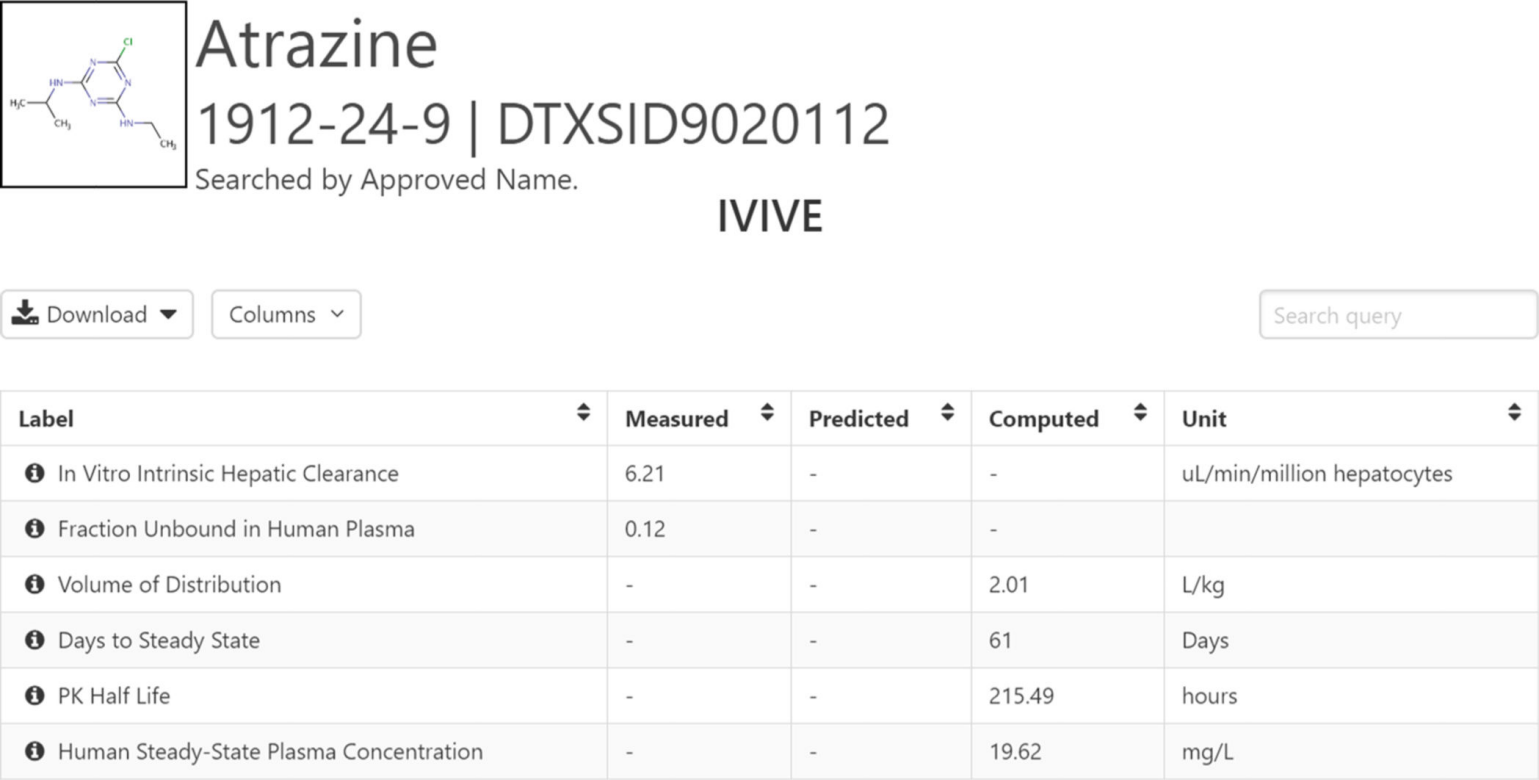
The IVIVE data associated with Atrazine. The measured data are the *in vitro* intrinsic hepatic clearance and fraction unbound (Fub) in human plasma. From these data, using an IVIVE algorithm (Bell, 2018), four other data types are computed - volume of distribution (Vd), days to steady state concentration, pharmacokinetic half-life and human steady-state plasma concentration. For details regarding a particular data type, hover over the informational icon.

Wetmore et al. (Wetmore, 2012) have reported measurement of hepatic metabolic clearance and plasma protein binding data for ca. 416 ToxCast chemicals. Historically, the data generation has been split into two phases, 238 phase 1 chemicals in 2012 (Chemicals, 2020) and 178 phase 2 chemicals in 2015 (Chemicals, 2020). and These datasets represent key determinants of *in vitro* chemical toxicokinetics and have been used to convert ToxCast bioactivity assays into daily human oral dose (also known as the oral equivalent dose) necessary to produce steady-state *in vivo* blood concentrations equivalent to *in vitro* concentrations at 50% of maximum activity (AC(50)) or lowest effective concentration values across more than 500 *in vitro* ToxCast assays. Such conversion has been performed using reverse dosimetry using the High (er) throughput toxicokinetics (HTTK) tool as a population-based *in vitro*-to-*in vivo* (IVIVE) extrapolation model which then allowed to compare these predictions with chemical intake estimates. Further refinements of HTTK (Wambaugh, 2015; Wambaugh, 2019) have recently allowed to quantify measurement uncertainty and biological variability into IVIVE using Monte Carlo simulations. Such uncertainty propagation methods allow integration of other sources of uncertainty such as *in silico* predictors of HTTK parameters (e.g., unbound fraction and intrinsic hepatic clearance). These approaches have the potential to inform risk-based prioritization based on the relationships between *in vitro* bioactivities and exposure estimates (Wambaugh, 2019). Both measured and predicted data may be available; the predicted data are available as a result of QSAR modeling. These data are accessible in the Dashboard under the ADME tab, for example, for Atrazine (Chemicals and Dashboard, 2020) (see Fig. 17).

## 14. Can I run QSAR predictions on the Dashboard to fill data gaps and download the results in Excel to aid my analysis?

**Fig. 18.**
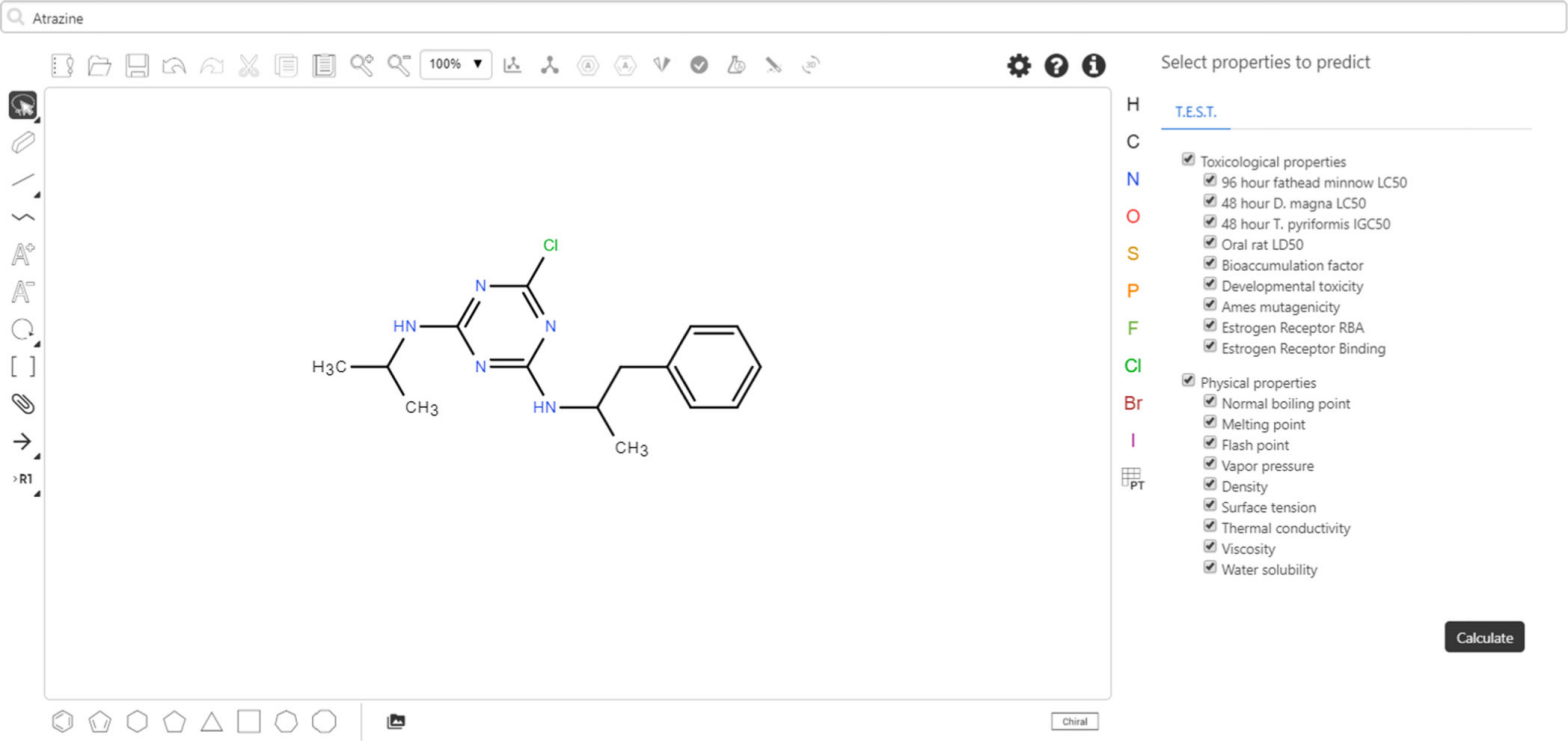
The chemical sketcher is initially populated with a search for “atrazine” and then the chemical structure is edited to give a chemical of interest, not in the database. The user clicks on the Calculate button to generate the various predictions for all selected endpoints.

Although chemical structures within the Dashboard have many physicochemical properties and fate and transport values predicted using one of the multiple prediction algorithms surfaced through the Dashboard, as described earlier (i.e., ACD/Labs, OPERA, TEST etc.), it is also possible to predict multiple endpoints (such as physicochemical, fate and transport, and toxicity data) via a newly implemented predictions interface (Chemicals, 2020). A user can draw a chemical using the chemical sketcher as shown in Fig. 18 and predict all endpoints served up through the Toxicity Estimation Software Tool (Tool, 2020). Alternatively, a user can use a chemical name or CAS RN to retrieve a chemical from the database and either predict on that chemical, or edit it into the form of the chemical of interest.

**Fig. 19.**
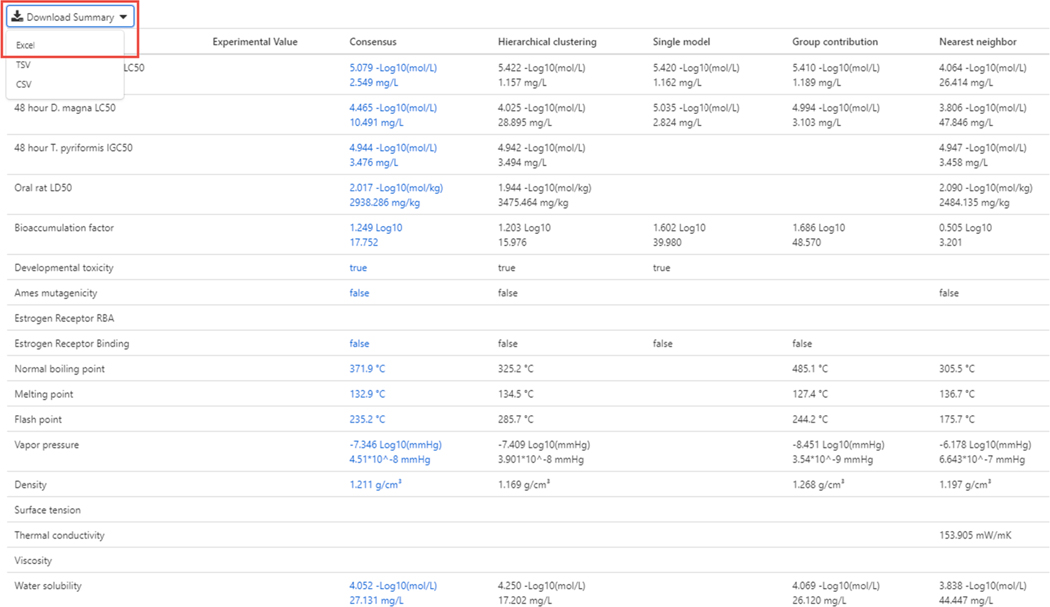
Four models are utilized to generate predictions for each of the endpoints. These are nearest neighbor, group contribution, single model and hierarchical clustering models. The consensus model is the average of all predictions. Notice that not all endpoints can be predicted by all models and that, in this case, there are **no** experimental data available for the chemical. If experimental data were available, it would be shown.

When the calculate button is selected, then four different modeling approaches are applied to generate predicted values and a consensus (average) value is provided (see Fig. 19). The results for all predictions can be downloaded as an Excel file, tab- or comma-separated file.

For each of the predictions, a detailed calculation report similar to that shown for pre-predicted data in the dashboard, generated in real time, is produced. For details regarding how to interpret the report, readers are referred to the TEST manual (User’s Guide for WebTEST (version 1.0) (Web-services Toxicity Estimation Software Tool), 2020).

## 15. Conclusions and future work

This article has presented some of the current capabilities of the Dashboard that we believe to be most useful and relevant to human health and ecological risk assessors. This includes the ability to extract data on chemical properties, *in vitro* and *in vivo* bioactivity data for hundreds to thousands of chemicals, as well as the use of *in silico* models to integrate NAMs for 21st century risk assessment. The tutorial covered the extraction of several types of chemical properties, including nomenclature and structure, HTS bioactivity concentration–response data from the ToxCast suite, *in vivo* dose–response data from DSSTox, and *in vitro* toxicokinetic data from HTTK. The use of *in silico* tools such as read-across and QSARs, available in GenRA, ACD/Labs, OPERA and TEST, as well as IVIVE models in HTTK as a means to convert bioactivity concentrations from *in vitro* assays into human equivalent doses through reverse dosimetry, is relevant to those tasked with evaluating chemicals for various decision contexts, such as risk assessment. The application of such approaches continue to be reported in peer-reviewed publications from our laboratory (Science, 2021) but a recent example is emphasized here. Paul-Friedman et al (Paul Friedman, 2020) have reported on how *in vitro* bioactivity can be used as a lower bound estimate of *in vivo* adverse effect levels to support risk-based prioritization, They compared PODs from high-throughput predictions of bioactivity, exposure predictions, and traditional hazard information for 448 chemicals and successfully demonstrated the feasibility of using *in vitro* bioactivity as a protective estimate of POD in screening-level assessments. It should be noted that when such studies are reported the Dashboard forms a pivotal role in ensuring that all chemicals are registered and curated, that the associated bioactivity data and exposure predictions are available, and, in this example, that the relevant chemical collection is available from the Dashboard as a list (Chemicals and Dashboard, 2020). An example of where the Dashboard provided direct support for risk assessment case studies of food additives has been reported by Turley et al. (Turley, 2019)Whereas this tutorial has focused primarily on the analysis of data associated with single chemicals, the batch search capability (Chemicals, 2020) provides the ability to access many of the data streams available in the Dashboard *en masse*; supporting inputs for up to 5000 CAS RNs or names, for example, and harvest the data into a downloadable format such as Excel, tab- or comma-separated or SDF file. Further details regarding batch search are described in more detail elsewhere (Lowe and Williams, 2021).

Other capabilities have been previously reported, including the use of the Dashboard to support mass-spectrometry analyses, especially non-targeted analysis (Ulrich, 2018; Chao, 2020; McEachran, 2020; Sobus, 2017; Newton, 2018; Sobus, 2019; Williams and Sobus, 2020; McEachran, 2019). In addition, new functionality is already under development in prototype form or planned for future iterations of the Dashboard. This includes the integration of *additional* real-time prediction models into the interface. At present real time calculations are limited only to TEST QSAR property and toxicity endpoint predictions, but future plans include integration of both EPI Suite and OPERA property predictions. The ToxValDB hazard database continues to expand with new data with every release of the Dashboard, but these data require manual curation review which will continue on an ongoing basis. The most highly curated *in vivo* data in the Dashboard, included in the ToxValDB database under the Hazard tab, are from the ToxRefDB database (Martin, 2009; Watford, 2019). These data are supplied only in simplified formats, currently, but richer visualizations of the data facilitating deeper data exploration are planned.

In addition, a public database of chemical time-series concentration data (concentration vs. time (CvT)) has been published very recently and includes data from 567 studies in humans and test animals (rat, mouse, dog, non-human primate) for 144 environmental chemicals and their metabolites (187 analytes total) (Sayre et al., 2020). The motivation in constructing the CvT database was to provide *in vivo* TK data for external validation and uncertainty analysis of *in vitro* assays and *in silico* models as NAMs to depict chemical’s toxicokinetics and determine internal concentrations at which bioactivity may occur. Future inclusion of the CvT database in the Dashboard is envisaged and will support assessment of the relationships between external doses and internal tissue exposures for chemical risk assessment. Other applications of the CvT database include the analyses of differential distribution across chemicals, species, doses, or routes, and *meta*-analyses on pharma- and toxicokinetic studies.

An important aspect to optimize the use of NAMs in human health risk assessment is to provide the opportunity to the risk assessment community to share data and models using harmonized formats and modelling procedures including transparent reporting of results, respectively. In this context, OECD has developed Harmonized Templates (OHTs) (Harmonised Templates, 2020) providing standardized data formats to report chemical properties for storing data regarding use and exposure and to help determine their potential hazard to human health and the environment. Such OHTs provide a means for governments and industry to facilitate the electronic exchange of test study summary information such as physicochemical properties (OHTs 1, to 23–5 & 101 to 113) (Harmonised Templates, 2020) and health effects including toxicokinetic information, acute, sub-chronic and chronic toxicity (OHT 58 to 84 & 86) (Harmonised Templates, 2020) for any regulated chemicals (e.g., pesticides, biocides, industrial chemicals, food/feed additives etc). Such exchange is further facilitated by the OECD eChemPortal (OECD eChemPortal: Global Portal to Information on Chemical Substances, 2020) as a worldwide open repository of chemical properties for which data are structured using OHTs and can be visualized and submitted via the IUCLID (IUCLID: International Uniform ChemicaL Information Database software, 2020) software, the latter used in the implementation of various regulatory programs such as the OECD Cooperative Chemicals Assessment Programme (CoCAP) (OECD Cooperative Chemicals Assessment Programme (CoCAP), 2020) and the EU REACH legislation (ECHA REACH, 2020).

Over the last decade, the OHT 201 template (Harmonised Template, 2020), providing a standard to collect non-apical observations obtained from methods such as *in vitro* testing or from other classes of methods (e. g., *ex vivo* or *in silico* methods), has been developed to provide mechanistic information underpinning Adverse Outcome Pathways (AOPs), i. e. effects on molecular, subcellular, cell, tissue or organ level. Such mechanistic information is of high relevance to hazard assessment through defined approaches or Integrated Approaches on Testing and Assessment (IATA) (OECD: Integrated Approaches to Testing and Assessment (IATA), 2020) and can be integrated through a weight-of-evidence approach. The latest version of OHT 201 (December 2020) further highlights the potential of the template to further harmonize the reporting and sharing of mechanistic information across scientific advisory bodies. These include several international organizations such as the World Health Organisation (WHO), the OECD, the Food and Agriculture Organisation (FAO), the US-EPA, the Food and Drug Administration (FDA), the European Chemicals Agency (ECHA), Health Canada, the Joint Research Center (JRC) of the European Commission, and the European Food Safety Authority (EFSA), as well as industry and major stakeholders, and will ultimately support the integration of NAMs in human and environment risk assessment. Data extracted from OHTs for chemicals, whether sourced through IUCLID or eChemPortal, will ideally be incorporated into the Dashboard in future versions.

As described in a recent article by Thomas et al. (Thomas, 2019) regarding future directions for computational toxicology at the U.S. EPA, there will be an ongoing expansion of data types in the future with the center for computational toxicology and exposure already examining the impact of xenobiotic metabolic processes on *in vitro* assay data (Deisenroth, 2020), the benefits of high-throughput transcriptomics (Harrill, 2019) and high-throughput phenotypic profiling data (Thomas, 2019). These data will ultimately need to be supported in future iterations of the Dashboard.

The Dashboard, from its humble beginnings as a proof-of-concept application, has had ten incremental releases since its first release in March 2015. With each release, the diversity of supported data has expanded with the functionality and it has become the primary application supporting the EPA’s computational toxicology research programs. It has, however, reached the maximum of its potential using the existing software architecture. The 10th, and final version, of the existing architecture underpinning the dashboard was released to the community in July 2020 and the data are not being updated further until the next release scheduled for Autumn 2021, which will utilize a new data-hub-based architecture and user-interface design. Moving to this new architecture will enable further growth and expansion of the Dashboard in support of chemical risk assessment activities.

## Supplementary Material

S1

S2

## Figures and Tables

**Fig. 16.**
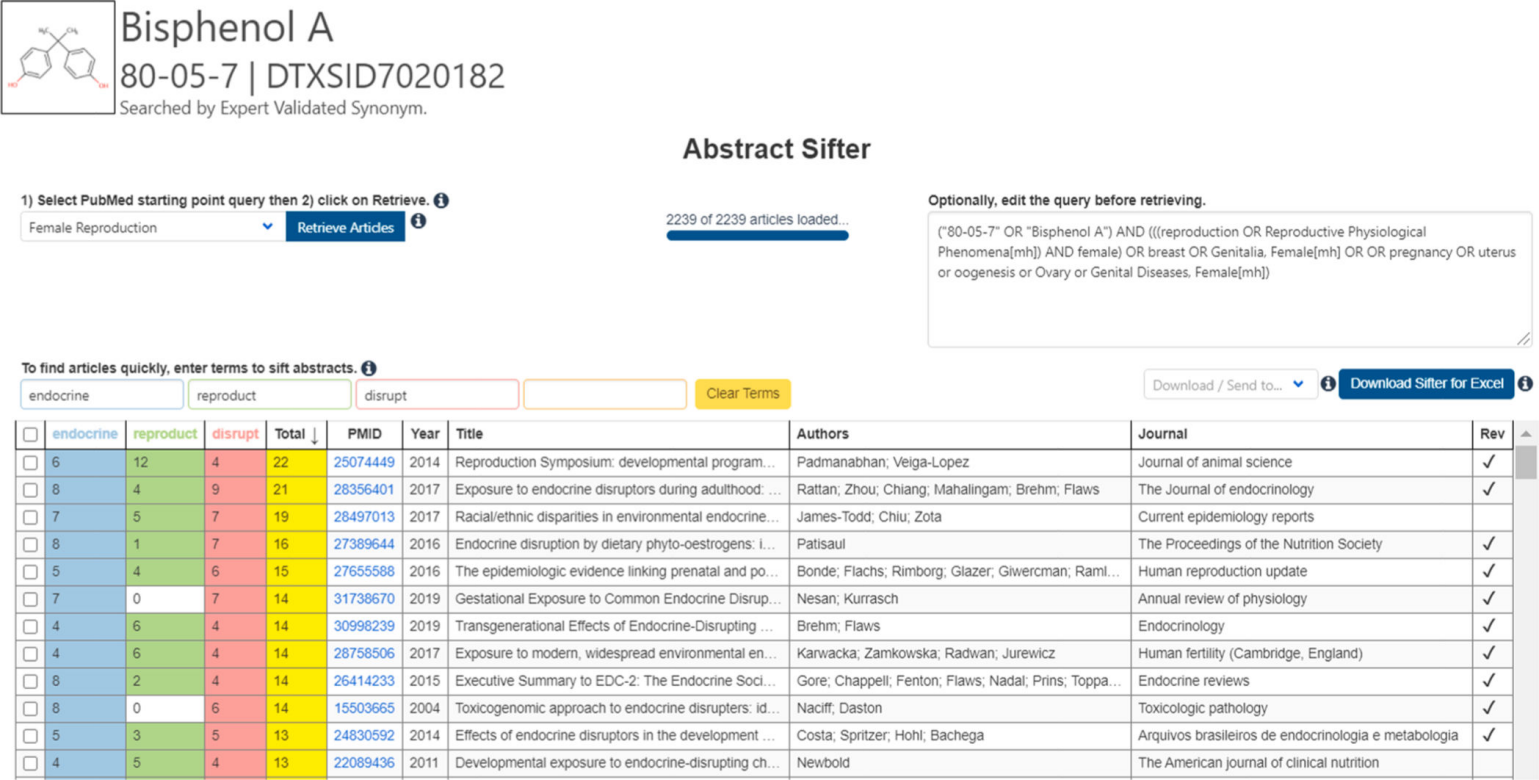
Abstract Sifter interface showing articles associated with the query “female reproduction” and sifted using three terms: endocrine (blue), reproduc (green) and disrupt (red) with the total count shown in yellow. The sifter interface includes PubMed IDs hyperlinked to PubMed and information associated wth articles: year of publication, title, author(s), journal and whether or not the article is a review. (For interpretation of the references to color in this figure legend, the reader is referred to the web version of this article.)

**Table 1:** Text strings associated with searching PubMed and Web of Science.

PubMed	(90–15–3[rn] OR “1-Naphthol”[tw] OR “Naphthalen-1-ol”[tw] OR “1- Naphthalenol”[tw] OR “1- naphthalenol”[tw])	Date: 07/31/2019 Results: 1,421

Web of Science	TS=(“1-Naphthol” OR “Naphthalen-1-ol” OR “1-Naphthalenol” OR “1-naphthalenol”)	Date: 07/31/2019Results: 2,640
**Total Results**	**Across all databases after duplicate removal**	**Results: 3,672**
